# Association between serum osmolality trajectories and mortality in sepsis patients: a retrospective multi-cohort study

**DOI:** 10.1186/s40001-025-03699-6

**Published:** 2025-12-25

**Authors:** Kun Fang, Jiacheng Shen, Bangchuan Hu, Ziqiang Shao, Li Li

**Affiliations:** 1 Zhejiang University School of Medicine, Hangzhou, Zhejiang China; 2 Geriatric Medicine Centre, Department of Geriatric Medicine, Affiliated People’s Hospital, Zhejiang Provincial People’s Hospital, Hangzhou Medical College, Hangzhou, Zhejiang China; 3 Emergency and Critical Care Centre, Department of Critical Care Medicine, Affiliated People’s Hospital, Zhejiang Provincial People’s Hospital, Hangzhou Medical College, Hangzhou, Zhejiang China

**Keywords:** Sepsis, Serum osmolality, Mortality, Fluid management, Intensive care unit, Trajectory modeling

## Abstract

**Background:**

Sepsis commonly leads to fluid and electrolyte imbalances, often reflected in abnormal serum osmolality. Although static measurements of osmolality have been investigated, the prognostic significance of dynamic changes in serum osmolality over time remains poorly understood in patients with sepsis.

**Methods:**

We conducted a comprehensive analysis of 22,737 septic patients from the MIMIC-IV and eICU-CRD databases, with external validation performed using an independent cohort of 303 patients from Zhejiang Provincial People's Hospital. Latent class trajectory modeling was applied to identify distinct patterns of serum osmolality during the first 4 days in the ICU. The association between trajectory patterns and mortality was assessed using Cox proportional hazards regression. Mediation analysis was employed to explore potential biological mechanisms, while parametric g-formula simulations were used to evaluate the impact of trajectory-specific fluid management strategies.

**Results:**

Three distinct serum osmolality trajectories were identified: stable (ST), ascending (AS), and descending (DS). Compared to the ST group, both the AS and DS groups were associated with significantly higher 28-day mortality (HR 1.80, 95% CI 1.61–2.01 for AS; HR 1.83, 95% CI 1.62–2.06 for DS). Mediation analysis indicated that renal dysfunction (accounting for 11.16% of the total effect, *P* < 0.001) and cumulative positive fluid balance (11.39%, *P* < 0.001) partially mediated the observed associations. Parametric g-formula simulations revealed substantial heterogeneity in responses to fluid management across trajectory groups, with optimal fluid strategies varying significantly by trajectory pattern and patient characteristics.

**Conclusion:**

Dynamic serum osmolality trajectories are independent predictors of mortality in sepsis, with effects partially mediated through renal dysfunction and fluid imbalance. These findings support the implementation of trajectory-guided precision fluid therapy as a novel framework for individualized sepsis care, challenging one-size-fits-all approaches and offering evidence-based guidance for personalized treatment strategies.

**Supplementary Information:**

The online version contains supplementary material available at 10.1186/s40001-025-03699-6.

## Background

Sepsis is a life-threatening condition characterized by a dysregulated host response to infection, leading to progressive multiple organ dysfunction and high mortality among critically ill patients [1, 2]. A fundamental feature of sepsis is the disruption of cellular homeostasis, primarily driven by electrolyte imbalances and metabolic disturbances [3]. These pathophysiological alterations are closely linked to changes in serum osmolality—an essential indicator of the concentration of osmotically active solutes. Abnormal serum osmolality in septic patients not only reflects underlying physiological derangements but also actively contributes to cellular injury, thereby exacerbating organ failure and increasing the risk of death. Thus, understanding the dynamic relationship between serum osmolality and mortality may enhance prognostic accuracy and inform more effective clinical management strategies.

Given the established clinical relevance of serum osmolality in determining sepsis outcomes, previous studies have consistently demonstrated a strong association between osmolality levels and mortality risk. Analyses based on single admission measurements typically reveal a U-shaped relationship, with both hypo- and hyperosmolality linked to elevated mortality. For example, Liang et al. found that 28-day mortality was significantly higher in septic patients presenting with either elevated (> 303.21 mOsm/L) or reduced (≤ 285.80 mOsm/L) serum osmolality upon admission [4]. Similarly, Heng et al. reported increased mortality in patients with abnormal osmolality (< 290 mOsm/L or > 309 mOsm/L) and further observed that fluid therapy conferred survival benefits specifically in those with hyperosmolality [5]. However, single-timepoint assessments fail to capture the longitudinal evolution of serum osmolality during the acute phase of sepsis. Trajectory-based analysis, which examines temporal patterns over time, provides a more comprehensive representation of dynamic physiological changes and better accounts for the heterogeneity inherent in sepsis. Wu et al. identified distinct 72-h serum osmolality trajectories in patients with sepsis-associated encephalopathy (SAE), showing that stable low-level osmolality (285–295 mOsm/L) was associated with lower mortality, whereas marked fluctuations predicted worse outcomes [6]. Despite these findings, existing evidence remains constrained to isolated timepoints or narrowly defined subgroups (e.g., SAE), leaving the broader prognostic value of osmolality trajectories in the general sepsis population largely unexplored. Therefore, this study aims to identify distinct serum osmolality trajectory patterns during the first four days of ICU admission using latent class trajectory modeling, evaluate their association with 28-day mortality, and explore the underlying pathophysiological mechanisms that link these trajectories to clinical outcomes.

To elucidate the mechanisms linking osmolality trajectories to clinical outcomes, it is essential to examine how disturbances in serum osmolality affect organ function. Accumulating evidence demonstrates that both hyperosmolality and hypoosmolality independently increase the risk of acute kidney injury (AKI) in critically ill patients [7–10]. Serum osmolality plays a pivotal role in regulating fluid balance by governing transmembrane water shifts and maintaining intracellular homeostasis. In sepsis, a systemic inflammatory response frequently induces increased capillary permeability, enabling macromolecules—particularly albumin—to leak from the intravascular space into the interstitium, leading to hypoalbuminemia. As albumin is the primary determinant of plasma colloid oncotic pressure, its depletion disrupts the osmotic gradient between intravascular and interstitial compartments. This heightened permeability, coupled with reduced oncotic pressure, promotes fluid extravasation into the "third space", resulting in interstitial edema. Consequently, this process contributes to intravascular hypovolemia and impaired tissue perfusion in septic patients, complicating fluid resuscitation strategies and representing a key pathophysiological mechanism associated with the dynamic changes in serum osmolality examined in our study. While current mechanistic insights are largely derived from single-timepoint assessments, we hypothesize that distinct osmolality trajectories influence clinical outcomes through shared biological pathways. Therefore, our analysis will evaluate potential mediating factors—including markers of organ dysfunction and cumulative fluid balance—to identify the physiological mechanisms through which specific osmolality patterns modulate mortality risk.

Based on this mechanistic framework, we hypothesize that septic patients with distinct serum osmolality trajectories may benefit from individualized fluid management strategies, which are associated with better outcomes. To test this hypothesis, we will apply the parametric g-formula method for causal inference, enabling the simulation of hypothetical interventions—such as daily fluid balance adjustments—and their potential effects on 28-day mortality [11]. This comprehensive analytical approach aims to generate actionable evidence for optimizing fluid resuscitation in sepsis, deliver more precise, trajectory-informed treatment recommendations, and clarify the relationship between dynamic osmolality patterns and patient outcomes. By identifying trajectory-specific responses to fluid therapy, our study seeks to advance sepsis care through the development of a data-driven foundation for personalized fluid management, ultimately supporting evidence-based clinical decision-making.

## Materials and methods

### Data source

Participants were identified from the eICU Collaborative Research Database (eICU-CRD) version 2.0 (2014–2015) and MIMIC-IV version 3.0 (2008–2022), both hosted on PhysioNet [12, 13]. For external validation, we included sepsis patients diagnosed according to Sepsis-3 criteria admitted to the Intensive Care Unit (ICU) at Zhejiang Provincial People’s Hospital (ZPPH), with data collected between February 2020 and July 2023. The first author completed human research protection training and obtained authorized access to the databases (ID: 11383,210). All personal identifiers were removed in accordance with data use agreements to protect patient confidentiality. The study was approved by the Institutional Review Board (IRB) of Beth Israel Deaconess Medical Center; written informed consent was waived due to the use of de-identified data. For the ZPPH cohort, the study protocol was reviewed and approved by the local IRB and Ethics Committee (Approval No. 2020KY002), and written informed consent was obtained from participants or their legally authorized representatives. The study adhered to the ethical principles outlined in the Declaration of Helsinki and complied with all applicable local regulations.

### Study population

We included adult patients (age ≥ 18 years) who met the Third International Consensus Definitions for Sepsis and Septic Shock (Sepsis-3), defined as a Sequential Organ Failure Assessment (SOFA) score ≥ 2 with suspected or confirmed infection [2]. Exclusion criteria comprised age < 18 years, repeated ICU admissions, ICU length of stay < 24 h, fewer than four daily serum osmolality measurements, and baseline serum creatinine (SCr) > 4 mg/dL, end-stage kidney disease (ESKD), or maintenance kidney replacement therapy (KRT) [14]. Additionally, we excluded patients who underwent renal replacement therapy (RRT) on the first day of ICU admission.

### Main variables and outcome variable

The primary variable was daily serum osmolality measured during the first 4 days following ICU admission, calculated using the formula: [15].

$${\text{Osmolality }}\left( {{\mathrm{mOsm}}/{\mathrm{L}}} \right) \, = { 2 } \times \, \left( {{\mathrm{Na}}^{ + } + {\text{ K}}^{ + } } \right) \, + \, \left( {{\text{glucose }}/{ 18}} \right) \, + \, ({\mathrm{BUN}}/{ 2}.{8})$$

When multiple measurements were available on a given day, the mean value was used for analysis. We extracted demographic information, clinical characteristics, laboratory values, vital signs, and comorbidity data for covariate adjustment and subgroup analyses. Variables with more than 40% missing data were excluded from the analysis (the proportion of missing data for all variables is presented in Additional file 5, Fig. 1) [16, 17]. For remaining missing values, multiple imputation techniques were applied to generate unbiased estimates while preserving statistical power and maintaining the full sample size [18]. The primary outcome was 28-day mortality, defined as death from any cause within 28 days of ICU admission, with follow-up censored at either death or study completion, whichever occurred first.

**Fig. 1:**
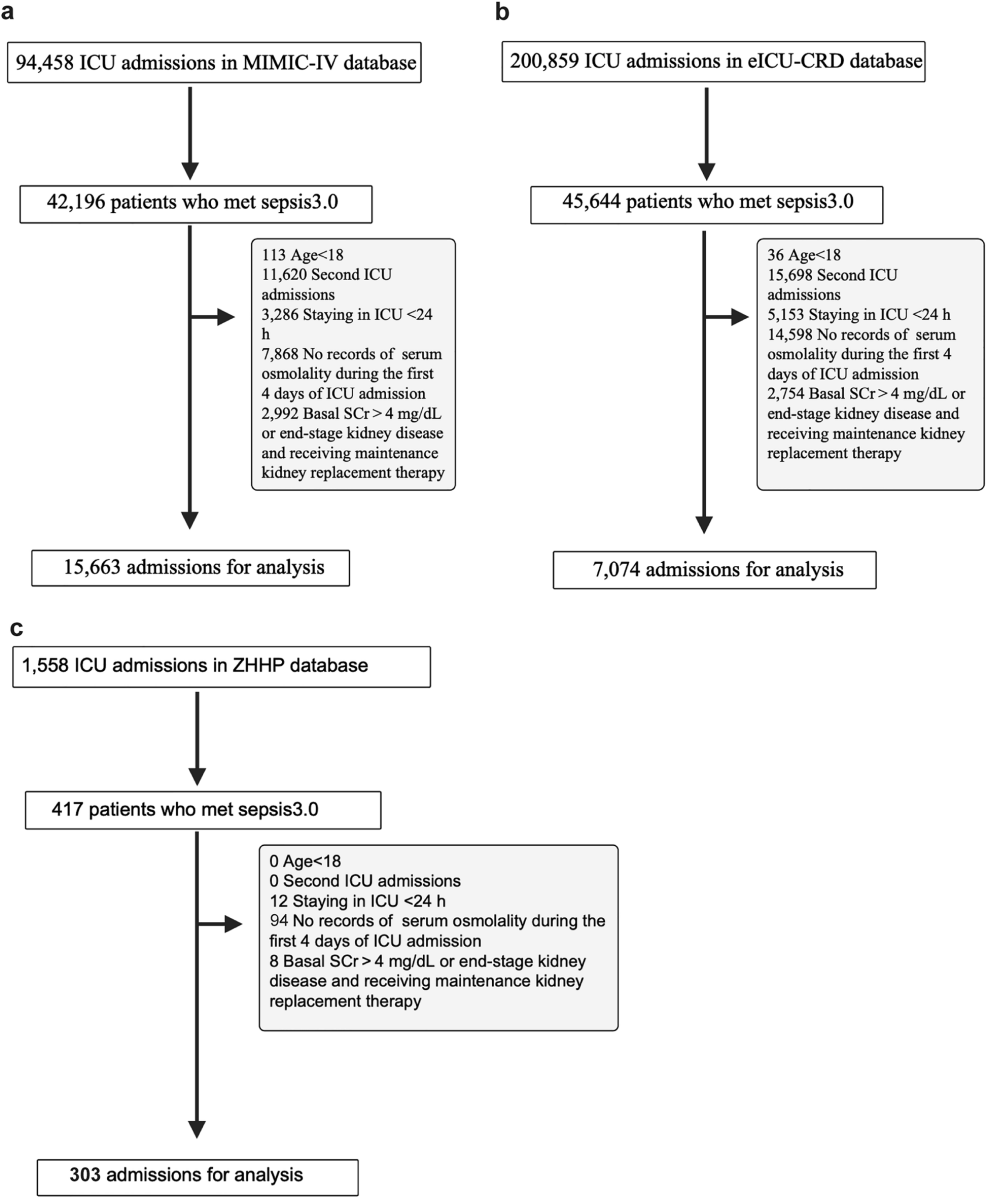
Cohort flow diagram of patient selection in three databases

### Serum osmolality dynamic trajectory identification

We employed latent class trajectory modeling (LCTM) to identify distinct subgroups of septic patients based on their serum osmolality dynamics during the first four ICU days [19]. This person-centered analytical approach classifies patients into latent subclasses with unique longitudinal trajectories using repeated osmolality measurements from the MIMIC-IV database. Model selection criteria included Akaike information criterion (AIC), Bayesian information criterion (BIC), and entropy values. To ensure clinical relevance, we required each subgroup to comprise ≥ 5% of the total sample. We subsequently validated these trajectory subgroups in both the eICU-CRD database and ZPPH cohort using the lcmm package (version 2.0.2) in R.

### Causal mediation analysis of dynamic trajectories and 28-day mortality

To minimize the risk of reverse causality, mediating variables were assessed from day 4 after ICU admission onward, covering the period through to the end of follow-up. Renal deterioration was defined as either new-onset AKI or progression of pre-existing AKI, according to the Kidney Disease: Improving Global Outcomes (KDIGO) criteria: an increase in serum creatinine ≥ 0.3 mg/dL within 48 h, a rise of ≥ 1.5-fold from baseline within 7 days, or urine output < 0.5 mL/kg/h for ≥ 6 consecutive hours [20]. Baseline creatinine was defined as the lowest recorded value within 7 days prior to AKI assessment. In the absence of pre-admission measurements, the initial ICU creatinine value was used as baseline. New-onset AKI referred to cases diagnosed after day 4 of ICU admission, while AKI progression was defined as an escalation in AKI stage during follow-up. Patients with pre-existing AKI at ICU admission or end-stage renal disease were excluded from mediator analysis [21]. Cumulative fluid balance was calculated as the net total of fluid intake minus output from day 4 until the end of follow-up. Finally, causal mediation analysis based on the VanderWeele framework was conducted to evaluate the indirect effects of osmolality trajectories on 28-day mortality mediated through these variables [22].

### Effect of daily fluid balance and the parametric G-formula

The parametric g-formula method was used to evaluate the impact of daily fluid balance on patient prognosis across different serum osmolality trajectory subtypes [11]. This approach accounts for time-dependent confounders by incorporating Monte Carlo simulation modeling. Intervention strategies were classified according to daily fluid balance (intake minus output) using the following thresholds: natural course (no intervention); aggressive negative (< − 3000 mL); moderate negative (− 3000 to − 2500 mL); mild negative (− 2500 to − 500 mL); conservative (− 500 to 500 mL); mild positive (500 to 2000 mL); moderate positive (2000 to 3000 mL); and aggressive positive (> 3000 mL) [23]. For simplified analysis, fluid balance was further categorized as negative (< − 500 mL), moderate (− 500 to 500 mL), or positive (> 500 mL). Covariate adjustments included baseline age. For variables with less than 50% missing data, only daily Sequential Organ Failure Assessment (SOFA) scores (after imputation) were included as time-varying covariates. Risk ratios (RR) with 95% confidence intervals (95% CI) were calculated to compare 28-day mortality between the natural course and each fluid management strategy.

### Statistical analysis

Categorical variables were presented as frequencies and percentages, while continuous variables were summarized as mean ± standard deviation or median (interquartile range), depending on data distribution. Group comparisons were conducted using Chi-square tests for categorical variables, one-way ANOVA for normally distributed continuous variables, and Kruskal–Wallis *H* tests for non-normally distributed data. Survival differences across osmolality trajectory patterns were evaluated using multivariable Cox proportional hazards models, with Kaplan–Meier curves and log-rank tests used to visualize and compare survival probabilities. Given the smaller sample size in the external validation cohort, accelerated failure time (AFT) modeling was employed as an alternative when the proportional hazards assumption was violated, thereby improving analytical robustness. Feature selection was performed through univariate Cox regression followed by multivariate stepwise selection and LASSO regression with tenfold cross-validation. Model robustness was assessed using five progressively adjusted models:

- *Model 1* unadjusted.
- *Model 2* adjusted for demographics (age, sex, Body Mass Index [BMI], Charlson Comorbidity Index [24], and pre-ICU hospital stay).
- *Model 3* additionally adjusted for physiological parameters (respiratory rate, oxygen saturation, and urine output).
- *Model 4* further adjusted for clinical interventions (vasopressor use and mechanical ventilation on ICU day 1).
- *Model 5* comprehensively adjusted for all above covariates plus laboratory parameters (anion gap, bicarbonate, creatinine, partial pressure of carbon dioxide, and prothrombin time).

Subgroup analyses were conducted to evaluate the consistency of osmolality trajectory effects across baseline characteristics, comorbidities, and treatment interventions. Results from the two primary cohorts (MIMIC-IV and eICU-CRD) were pooled using fixed-effects meta-analysis, with interaction *P*-values reported to assess heterogeneity. All statistical analyses were performed in R software (version 4.2.3), and a two-sided *P*-value < 0.05 was considered statistically significant.

## Results

### Serum osmolality trajectories and baseline characteristics

**Fig. 2:**
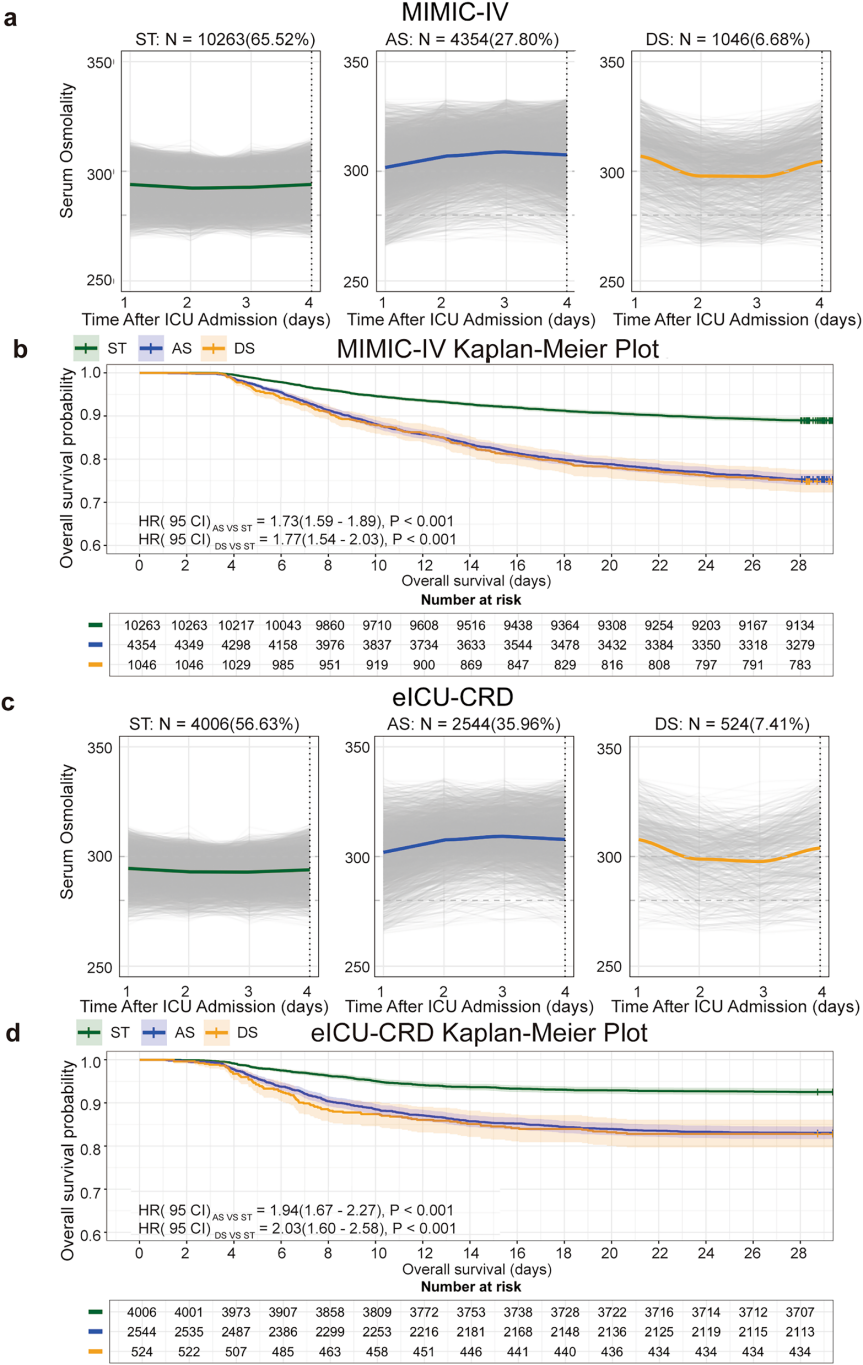
Trajectory and Kaplan–Meier plots of sepsis patients with different serum osmolality trajectories in two databases. Serum osmolality patterns for stable (ST), ascending (AS), and descending (DS) trajectories in MIMIC-IV (**a**) and eICU-CRD (c) databases. Kaplan–Meier survival curves for MIMIC-IV (**b**) and eICU-CRD (**d**) patients. Hazard ratios (HR) and *P*-values are shown for AS vs. ST and DS vs. ST comparisons

This study included 23,040 patients: 15,663 from the MIMIC-IV development cohort, 7074 from the eICU-CRD validation cohort, and 303 from the external validation cohort (ZPPH ICU) (Fig. 1). Using latent class trajectory modeling (Additional file 1), serum osmolality trajectories during the first 4 days in the ICU were classified into three distinct patterns:

- *Stable (ST)* osmolality remained within normal levels (285–300 mOsm/L).
- *Ascending (AS)* progressive increases in osmolality.
- *Descending (DS)* a decline from elevated to normal levels (Fig. 2a, c).

Baseline characteristics are summarized in Table 1. The MIMIC-IV and eICU-CRD cohorts exhibited comparable profiles, both dominated by the ST pattern (65.5% and 56.6%, respectively), with moderate disease severity. In stark contrast, the external ZPPH cohort (*N* = 303) presented a significantly higher burden of critical illness. Despite a similar median age, ZPPH patients had markedly higher baseline SOFA scores (median: 10.0 vs. 5.0 in MIMIC-IV and 6.0 in eICU-CRD) and SAPS II scores (median: 48.0 vs. 38.0 in MIMIC-IV and 46.0 in eICU-CRD) . This increased severity was consistent with a dramatic shift in osmolality phenotypes: the AS trajectory became the predominant pattern (66.0%), while the ST group accounted for only 19.8%. Consequently, ZPPH patients required substantially more intensive life support, as evidenced by greater use of vasopressors, dialysis, and mechanical ventilation (69.3% overall utilization compared to 64.5% in MIMIC-IV and 59.7% in eICU-CRD).

**Table 1:** Baseline characteristics of patients in three databases

Characteristics	MIMIC-IV (*N* = 15,663)	eICU (*N* = 7074)	ZPPH (N = 303)
Demographics			
Age, years	68.4 [57.2, 79.1]	66.0 [55.0, 77.0]	69.0 [60.5;79.0]
Male sex	8848 (56.5%)	3776 (53.4%)	207 (68.3%)
Admission type			
Medical	10,915 (69.70%)	6177 (87.30%)	225 (74.30%)
Surgical/trauma	4748 (30.30%)	897 (12.70%)	78 (25.70%)
Class			
AS	4354 (27.8%)	2544 (36.0%)	200 (66.0%)
DS	1046 (6.7%)	524 (7.4%)	43 (14.2%)
ST	10,263 (65.5%)	4006 (56.6%)	60 (19.8%)
CCI score	4.00 [2.0, 4.0]	5.00 [3.0, 7.0]	1.00 [0.0;1.0]
Severity scores			
SOFA score	5.0 [3.0, 7.0]	6.0 [4.0, 9.0]	10.0 [7.0, 13.0]
SAPS II score	38.0 [30.0, 46.0]	46.0 [36.0, 59.0]	48.0 [40.0;54.0]
Treatment supports			
MV, Day 1	8992 (57.4%)	3763 (53.2%)	193 (63.7%)
Vasopressors, Day1	4229 (27.0%)	845 (11.9%)	212 (70.0%)
MV during ICU Stay	10,106 (64.5%)	4225 (59.7%)	210 (69.3%)
Vasopressors during ICU stay	5288 (33.8%)	1114 (15.70%)	236 (77.9%)
Dialysis during ICU Stay	549 (3.51%)	188 (2.7%)	157 (51.8%)
MV duration, days	0.6 [0.0, 3.1]	0.0 [0.0, 2.0]	0.0 [0.0, 10.0]
MV delay	2748 (17.5%)	814 (11.50%)	32 (10.56%)
Max NE dose, Day1 (μg/kg/min)	0.0 [0.0, 0.1]	0.0 [0.0, 0.0]	0.0 [0.0, 0.1]
Max NE dose, Total (μg/kg/min)	0.0 [0.0, 0.2]	0.00 [0.0, 0.0]	0.0 [0.0, 0.1]
Nutrition route			
Enteral	4959 (31.7%)	1117 (15.8%)	97 (32.0%)
Parenteral	517 (3.3%)	401 (5.7%)	13 (4.3%)
Fluid management (mL)			
Input, Day 1	3097 [1729, 4723]	4827 [0, 15561]	3208 [1213, 6129]
Output, Day 1	2030 [1140, 3145]	5489 [2809, 10394]	2545 [1222;4436]
Balance, Day 1	738 [− 188.0, 2349.0]	− 342.3[− 3549.4, 6108]	584 [− 773.86, 3004]
Input, Day 2	1857 [820, 3099]	671 [0.00, 4408]	1845 [267;3316]
Output, Day 2	1500 [725, 2486]	2075 [550, 4425]	1625 [540, 2808]
Balance, Day 2	35.7 [− 472.8, 1218]	− 227.0[− 1974.8, 540]	0.0 [− 481.2, 1051]
Input, Day 3	1206 [0.0, 2518]	169 [0.0, 3257]	1178 [0.0, 2611]
Output, Day 3	1167 [0.0, 2390]	1650 [0.0, 4198]	1225 [0.0, 2498]
Balance, Day 3	0.0 [− 431.64, 485]	0.00 [− 1926.5, 15.0]	0.00 [− 612.5, 344]
Input, Day 4	520 [0.0;2128]	0.00 [0.0, 1926]	100 [0.0, 2019]
Output, Day 4	498 [0.0, 2140]	971 [0.0, 3450]	500 [0.0, 2478]
Balance, Day 4	0.0 [− 197.1, 50.8]	0.0 [− 1609.5, 0.0]	0.0 [− 532.5, 0.0]
Balance, Day 4–28	0.0 [− 530.0, 0.0]	0.0 [− 6936.3, 0.0]	0.0 [− 1901.1, 0.0]
Outcomes			
ICU LOS, days	4.0 [2.2, 7.9]	4.0 [2.0, 7.0]	12.0 [7.0, 21.0]
Mortality, 28-day	2467 (15.8%)	820 (11.6%)	120 (39.6%)

*AS/DS/ST* ascending/descending/stable, *CCI* Charlson comorbidity index, *SOFA* Sequential Organ Failure Assessment, *SAPS II* Simplified Acute Physiology Score II, *MV* mechanical ventilation, *ICU* intensive care unit, *NE* norepinephrine, *LOS* length of stay

Data are presented as No. (%) for categorical variables and median [interquartile range] for continuous variables, unless otherwise indicated

Distinct temporal patterns in fluid management were observed across cohorts. During the initial resuscitation phase (Day 1), fluid intake was substantial in all groups (median: 3097–4827 mL), resulting in positive net balances in MIMIC-IV (+ 738 mL) and ZPPH (+ 584 mL), whereas the eICU-CRD cohort exhibited a negative balance due to high output. As the clinical course progressed (Days 2–4), fluid administration decreased significantly, with daily balances trending toward neutrality. By the later phase (Days 4–28), the ZPPH cohort demonstrated a cumulative tendency toward negative fluid balance (IQR: − 1901 to 0 mL), consistent with a transition to de-resuscitation or stabilization. A comprehensive list of all baseline characteristics and daily fluid balance measurements is provided in Additional file 2.

### Serum osmolality trajectory patterns and 28-day mortality

Kaplan–Meier survival analysis revealed significant differences in 28-day mortality across osmolality trajectory groups in both the MIMIC-IV and eICU-CRD cohorts (Fig. 2b, d). In the MIMIC-IV cohort, compared to patients with ST trajectories, those with AS patterns had a higher risk of mortality (HR = 1.73, 95% CI 1.59–1.89, *P* < 0.001), while patients with DS patterns showed a similarly elevated risk (HR = 1.77, 95% CI 1.54–2.03, *P* < 0.001). These results were consistently observed in the eICU-CRD cohort: AS patients exhibited an HR of 1.94 (95% CI 1.67–2.27, *P* < 0.001); DS patients had an HR of 2.03 (95% CI 1.60–2.58, *P* < 0.001).

**Fig. 3:**
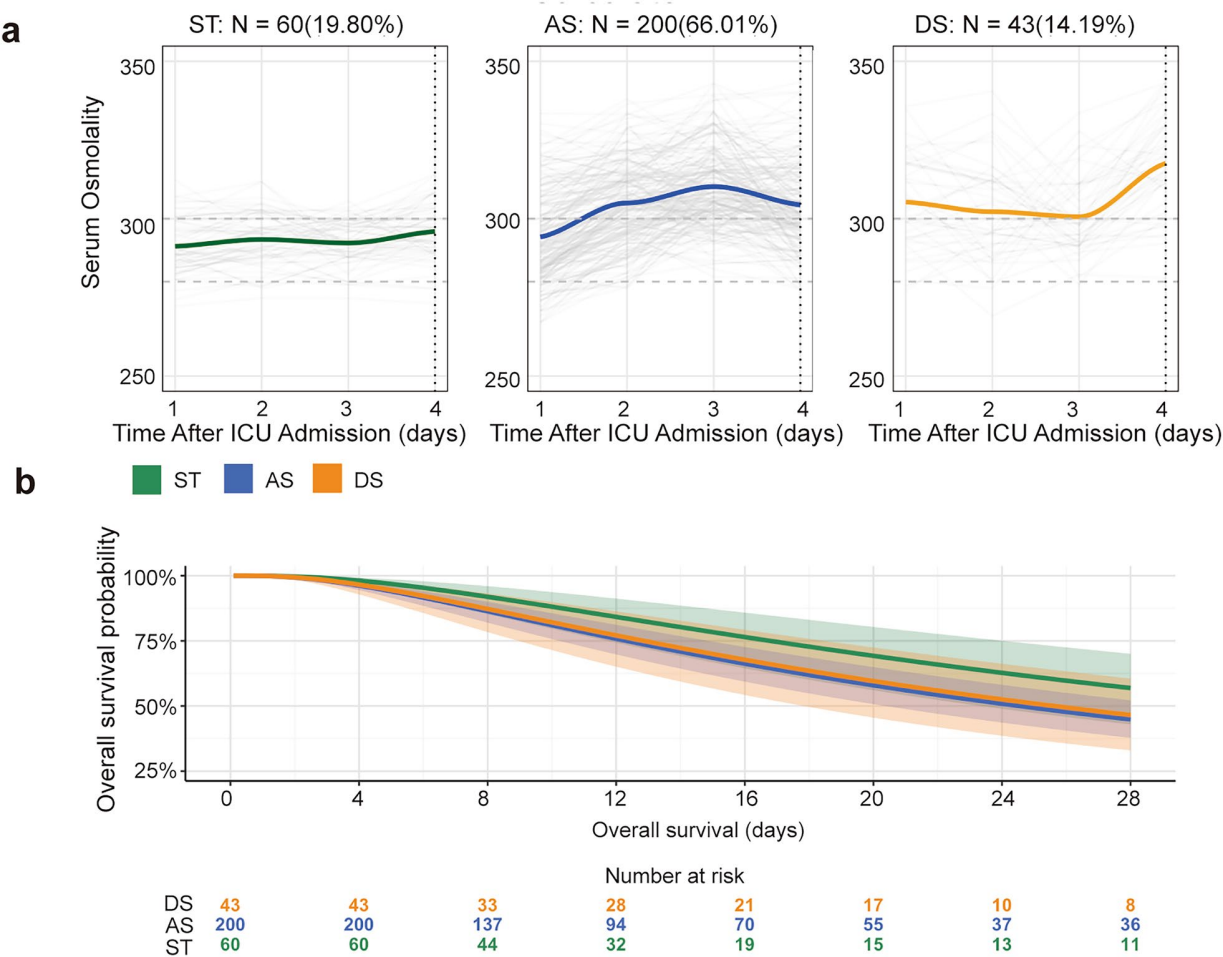
External validation of trajectory model and survival outcomes. Validation of trajectory patterns: ST, 19.80%; AS, 66.01%; DS, 14.19% (**a)**. Accelerated failure time survival analysis demonstrating differential 28-day mortality across trajectory groups (**b**)

The external validation cohort further confirmed these associations using accelerated failure time (AFT) modeling, demonstrating significantly worse outcomes for both DS and AS patients relative to ST patients (Fig. 3b). Multivariable sensitivity analyses supported the robustness of these findings (Additional file 3). In the fully adjusted model, meta-analysis of the two primary cohorts yielded pooled HRs of 1.80 (95% CI 1.61–2.01, *P* < 0.001) for AS patients and 1.83 (95% CI 1.62–2.06, *P* < 0.001) for DS patients.

### Subgroup analysis of associations between trajectory patterns and 28-day mortality

**Fig. 4:**
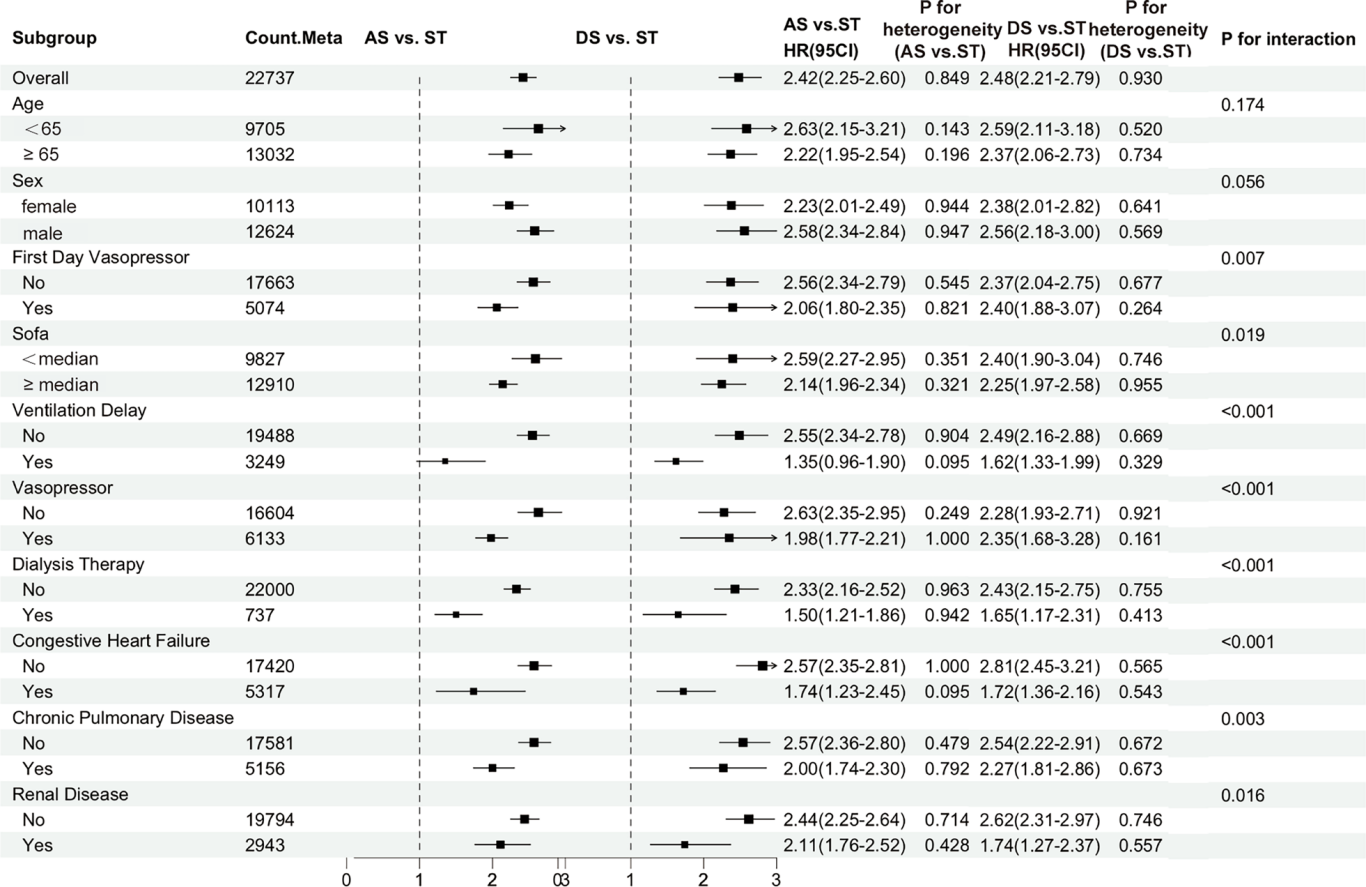
Subgroup meta-analysis of osmolality trajectory impacts on 28-day mortality in MIMIC-IV and eICU-CRD databases. Forest plot showing HR and 95%CI for AS and DS patterns compared to ST pattern across clinical subgroups (pooled *N* = 22,737). *P*-values for interaction between subgroups and osmolality trajectory effects are shown. Subgroup definitions: SOFA score was dichotomized at themedian value (5 in MIMIC-IV, 6 in eICU-CRD)

Stratified analyses were conducted across patient subgroups defined by demographics, disease severity, ICU interventions, and comorbidities, including 22,737 patients from the MIMIC-IV and eICU-CRD databases (Fig. 4 and Additional file 5. Figure 2). Compared to the ST trajectory pattern, both AS and DS patterns consistently showed an increased risk of 28-day mortality across most subgroups. The HRs for the AS pattern ranged from 1.35 to 2.63, while those for the DS pattern ranged from 1.62 to 2.81. Significant effect modification was observed in several subgroups, including first-day vasopressor use (*P* = 0.007), SOFA score categories (*P* = 0.019), ventilation delay (*P* < 0.001), vasopressor requirement within ICU stay (*P* < 0.001), dialysis therapy within ICU stay (*P* < 0.001), congestive heart failure (*P* < 0.001), chronic pulmonary disease (*P* = 0.003), and renal disease (*P* = 0.016).

### Mediating analysis between serum osmolality trajectory and 28-day mortality

Causal mediation analysis revealed that renal deterioration significantly mediated the association between serum osmolality trajectory and 28-day mortality, accounting for 11.16% of the total effect (indirect effect HR: 1.07, 95% CI 1.04–1.11) in the combined MIMIC-IV and eICU-CRD cohorts (Table 2). Additionally, cumulative positive fluid balance also demonstrated significant mediating effects, contributing 11.39% of the total effect (indirect effect HR: 1.07, 95% CI 1.04–1.11), with consistent mediation patterns observed across both databases.

**Table 2:** Causal mediation analysis of osmolality trajectories on 28-day survival within 4–28 days after ICU admission

Variable of mediation	Database	Average direct effect	*P*	Average causal mediate effect	*P*	Proportion mediated
		HR (95% CI)		HR(95% CI)		%
Renal deterioration	MIMIC-IV	1.75 (1.64–1.82)	< 0.001	1.24 (1.15–1.34)	< 0.001	27.56%
	eICU-CRD	1.74 (1.57–1.91)	< 0.001	1.04 (1.01–1.08)	0.035	6.40%
	Meta	1.75 (1.67–1.83)	< 0.001	1.07 (1.04–1.11)	< 0.05	11.16%
Cumulative positive fluid balance	MIMIC-IV	1.75 (1.65–1.83)	< 0.001	1.14 (1.10–1.21)	< 0.001	19.22%
	eICU-CRD	1.75 (1.60–1.93)	< 0.001	1.03 (0.99–1.08)	0.14	5.13%
	Meta	1.14 (1.10–1.21)	< 0.001	1.07 (1.04–1.11)	< 0.001	11.39%

Average direct and indirect effects of renal deterioration and cumulative positive fluid balance (4–28 days) on survival are presented using data from the MIMIC-IV and eICU-CRD databases. Effects are expressed as HR (95% CI) with *P*-values, along with the proportion mediated by each variable

### G-formula estimated risks of ICU 28-day mortality under different daily fluid balance management strategies for varying serum osmolality trajectories in MIMIC-IV and eICU-CRD

**Fig. 5:**
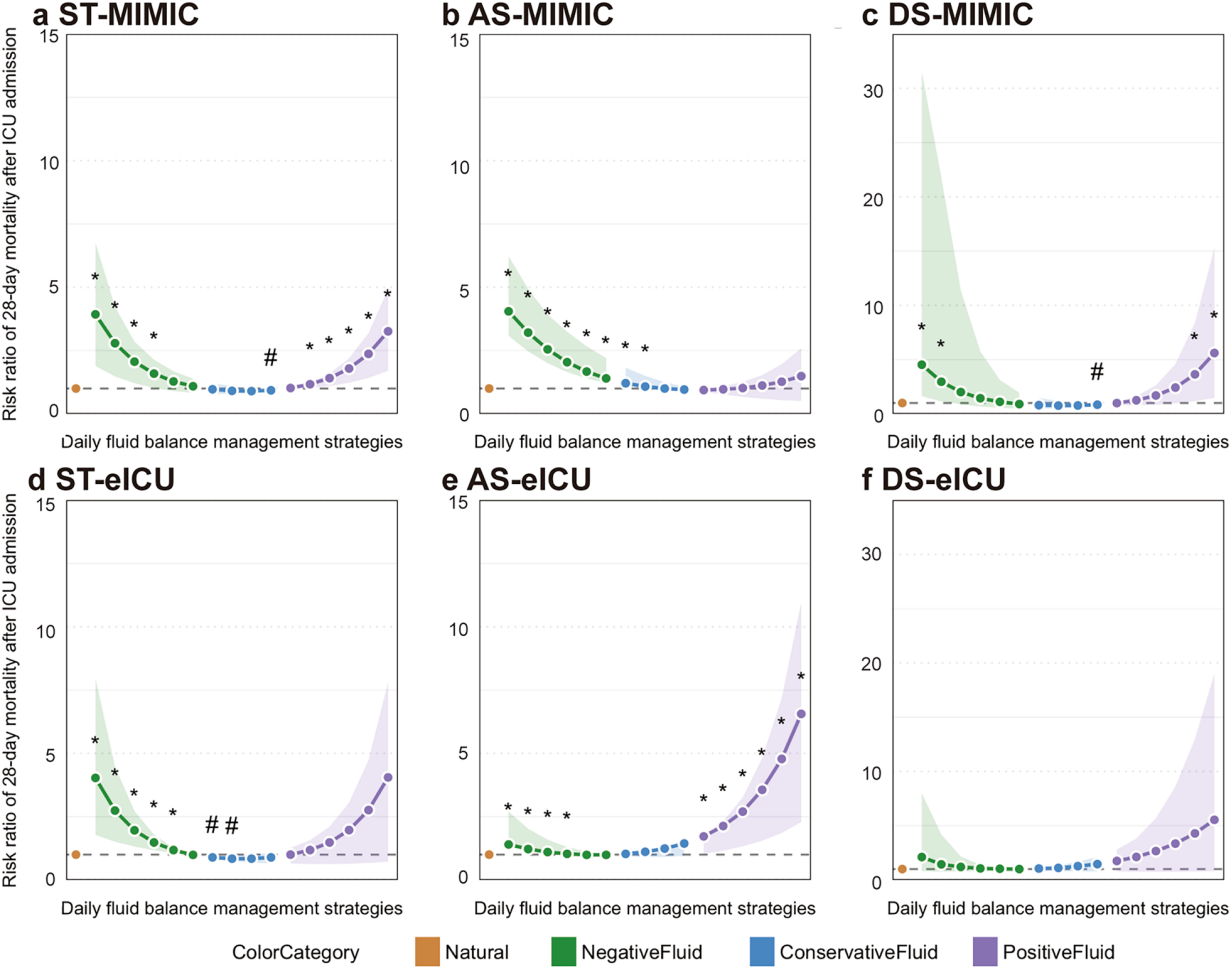
Daily fluid management and mortality across osmolality trajectories in two databases. ST, AS, and DS patterns are shown for both the MIMIC-IV (**a**–**c**) and eICU-CRD (**d**–**f**) cohorts. Color curves and bars represent fluid management strategies, with significance markers (* for worse outcomes, # for improved outcomes)

The g-formula analysis revealed significant heterogeneity in mortality risks across fluid management strategies in the MIMIC-IV and eICU-CRD cohorts (Fig. 5). Patients with the ST trajectory consistently benefited from conservative fluid management in both databases, as indicated by RR associated with positive fluid balance (peak RR: 3.26 in MIMIC-IV and 4.05 in eICU-CRD) (Additional file 4). The AS trajectory group exhibited the greatest inter-database variability, with patients in the eICU-CRD cohort showing markedly higher vulnerability to positive fluid balance (peak RR: 6.56 in eICU-CRD vs. 1.49 in MIMIC-IV). In contrast, patients with the DS trajectory displayed divergent responses: data from MIMIC-IV indicated a significant reduction in mortality with conservative negative fluid management (RR range: 0.76–0.84), whereas no such benefit was observed in the eICU-CRD cohort (RR range: 1.05–1.46). These findings highlight the importance of adopting trajectory-specific and context-sensitive fluid management strategies in critical care.

## Discussion

This study identified three distinct serum osmolality trajectories and evaluated their association with 28-day mortality in patients with sepsis. We demonstrated that these associations are mediated by renal deterioration and fluid imbalance, findings that support the development of targeted fluid management strategies. Notably, despite significant baseline heterogeneity—exemplified by the markedly higher disease severity in our local ZPPH cohort—the trajectory patterns and their prognostic significance remained highly consistent across diverse databases. This consistency underscores the robustness of our trajectory model as a reliable tool for risk stratification, while further analyses highlight the context-dependent nature of these associations.

Serum osmolality reflects blood solute concentration and serves as a crucial indicator for identifying high-risk patients with sepsis. While previous studies using static measurements have shown that both low (≤ 280 mOsm/L) and high (≥ 300 mOsm/L) osmolality levels are associated with increased mortality in critical illness [25–27], these single-timepoint assessments fail to capture the dynamic physiological changes that occur during sepsis progression. Our trajectory-based analysis revealed that patients with AS and DS osmolality patterns had significantly higher risks of 28-day mortality compared to those with ST trajectories. However, subgroup analyses identified significant interactions with key clinical variables, including delayed ventilation, vasopressor use, dialysis therapy, congestive heart failure, and chronic pulmonary disease. Notably, patients receiving intensive interventions or presenting with specific comorbidities exhibited a paradoxical reduction in mortality risk despite abnormal osmolality trajectories. These findings suggest that early changes in osmolality may reflect adaptive physiological responses to therapeutic interventions rather than purely pathological processes, highlighting the importance of clinical context in interpretation. Consequently, dynamic monitoring of serum osmolality provides valuable insights into sepsis pathophysiology and may support precision medicine approaches for individualized patient management.

To elucidate the mechanisms underlying the association between osmolality trajectories and mortality, we investigated biological pathways through which osmolality disturbances may drive multi-organ failure and transmembrane fluid shifts [28–30]. Our mediation analysis confirmed that unstable trajectories (AS and DS) increase mortality by exacerbating these pathological processes: specifically, renal deterioration mediated 11.16% of the total effect, and cumulative positive fluid balance mediated 11.39%, collectively accounting for approximately 22% of the overall risk. This mechanistic complexity is further illustrated by a distinct “terminal rebound”—a sharp rise in osmolality—observed in the DS trajectory around days 3–4, particularly in the ZPPH cohort. This late surge may reflect worsening renal function or iatrogenic effects of aggressive de-resuscitation (e.g., hemoconcentration), underscoring the need for further physiological investigation.

Building upon our mechanistic understanding of the association between osmolality trajectories and mortality, we recognize persistent clinical uncertainty regarding optimal fluid resuscitation strategies in sepsis management, which has led to substantial variation in clinical practice. While some clinicians advocate for high-volume fluid resuscitation, others favor more restrictive approaches, often administering volumes below the 30 mL/kg bolus recommended by the Surviving Sepsis Campaign [31]. Recent large-scale randomized controlled trials (RCTs) have highlighted the severe limitations of "one-size-fits-all" strategies, challenging the dogma surrounding both uniform resuscitation endpoints and fixed fluid volumes. For instance, the ANDROMEDA-SHOCK trial, which compared peripheral perfusion-targeted resuscitation with standard lactate-guided therapy, found no significant difference in mortality [32]. This outcome strongly suggests that a strategy based solely on lactate clearance may be suboptimal and could lead to unnecessary fluid administration. Similarly, universal volume-based strategies have shown limited efficacy. Both the CLASSIC trial (in the post-resuscitation phase) and the CLOVERS trial (in the early phase) compared restrictive versus liberal fluid strategies and likewise reported no significant mortality benefit for either approach [33, 34]. Collectively, these "negative" or "non-inferiority" findings provide robust evidence that sepsis is a highly heterogeneous syndrome, fundamentally undermining the "one-size-fits-all" paradigm by demonstrating that no single rigid protocol is universally optimal.

These inconclusive results have catalyzed a shift toward dynamic, trajectory-based management strategies. Supporting this paradigm shift, studies in other critical care populations—such as Beaubien-Souligny et al.'s analysis of patients with AKI—have shown that distinct longitudinal fluid balance patterns offer superior prognostic value compared to conventional static assessments, with specific trajectories strongly associated with improved survival outcomes [35].

**Fig. 6:**
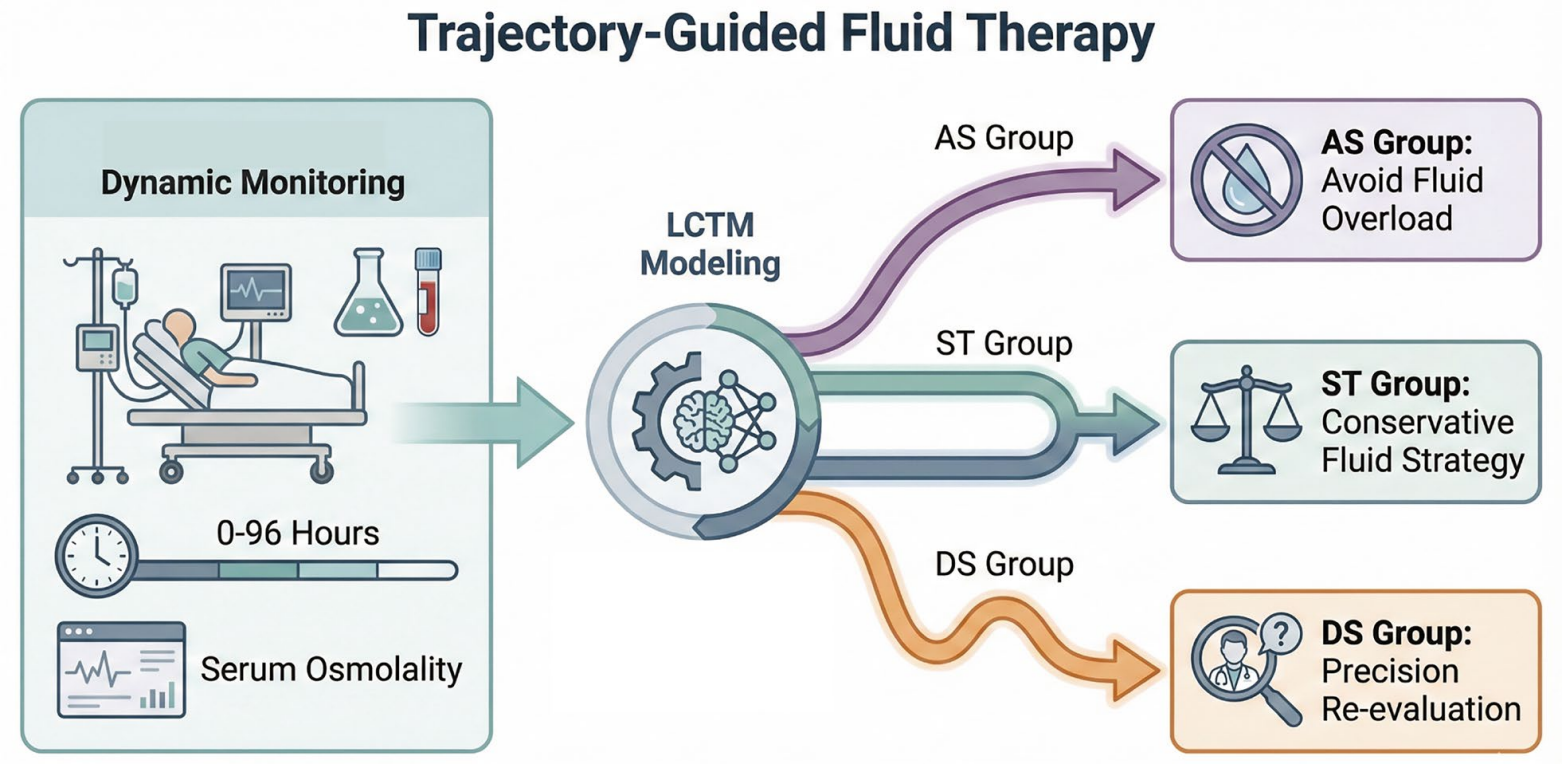
Conceptual framework of trajectory-guided fluid therapy

Building upon this evidence for dynamic assessment, we apply a longitudinal perspective to our findings to propose a trajectory-guided approach. To ensure reproducibility, we operationally define this approach as the classification of physiological phenotypes using LCTM of calculated serum osmolality measured during the first 96 h of ICU admission (Fig. 6). The rationale rests on the fundamental divergence in fluid responsiveness observed across trajectories. Specifically, the ST trajectory serves as a reliable reference, exhibiting a consistent U-shaped risk profile, which supports a strategy of "maintenance and homeostasis" through conservative fluid titration. In contrast, unstable trajectories require context-specific management protocols. The AS trajectory displays divergent risks—ranging from intolerance to fluid overload to sensitivity to negative fluid balance—and therefore supports a generally restrictive approach that prioritizes avoiding volume excess while preventing excessive dehydration. Meanwhile, the DS trajectory, characterized by narrow safety margins, necessitates a "precision re-evaluation" protocol involving frequent adjustments guided by real-time renal and perfusion markers to prevent both hypoperfusion and fluid congestion. This dichotomy—between the predictable physiology of ST patients and the complex, variable needs of AS and DS patients—demonstrates that optimal fluid management must be precisely tailored to the individual’s evolving physiological trajectory rather than adhering to a one-size-fits-all model [36].

Translating these findings into clinical practice, trajectory-guided therapy could serve as a novel dynamic assessment tool that complements existing sepsis bundles by using real-time monitoring of calculated serum osmolality to detect deviations from the ST trajectory—specifically early AS or DS patterns—and trigger timely adjustments toward conservative fluid strategies. However, feasible bedside implementation requires integrating longitudinal trajectory algorithms into electronic medical records (EMR) systems with clinical decision support (CDS) tools, as manual tracking of multi-day trends is impractical for clinicians in high-workload settings. Furthermore, interpretation of our results must account for limitations inherent to the retrospective design and the use of calculated rather than directly measured osmolality. Although calculated values have demonstrated a strong correlation with direct measurements (R values: 0.61–0.91; see Additional file 5. Figures 3, 4 and 5a-d), unmeasured solutes contributing to the osmolal gap may theoretically influence trajectory classification (median osmolality gap: − 5.33–6.10 mOsm/L; see Additional file 5. Figures 3, 4 and 5e). Additionally, the precise mechanistic interplay between renal impairment and fluid imbalance warrants further investigation. Given these implementation challenges and knowledge gaps, prospective multicenter studies across diverse global populations are needed to validate the robustness of these trajectories and assess the clinical utility of precision-based fluid management.

## Conclusions

This comprehensive analysis demonstrates that dynamic serum osmolality trajectories are powerful independent prognostic markers in sepsis patients. Unstable patterns (AS and DS) are associated with a significantly higher mortality risk compared to ST trajectories. Mechanistically, renal deterioration and cumulative positive fluid balance mediate a substantial proportion of the trajectory–mortality relationship, confirming that osmolality fluctuations exacerbate organ dysfunction through established pathophysiological pathways.

Crucially, trajectory-specific fluid management analysis reveals significant heterogeneity in treatment response across osmolality patterns, challenging the validity of universal sepsis resuscitation strategies. These findings establish trajectory-guided precision fluid therapy as a novel framework for individualized sepsis treatment. This approach provides clinicians with practical guidance for optimizing real-time fluid management decisions, thereby advancing evidence-based care in critically ill patients.

## Supplementary Information

Supplementary material 1.

Supplementary material 2.

Supplementary material 3.

Supplementary material 4.

Supplementary material 5.

## Data Availability

The datasets(MIMIC-IV and eICU-CRD) analysed during the current study are available in the PhysioNet (https://physionet.org). The ZPPH cohort data that support the findings of this study are not openly available due to reasons of sensitivity and are available from the corresponding author upon reasonable request.

## Abbreviations

SOFA: Sequential organ failure assessment
SAPS II: Simplified acute physiology score II
eICU-CRD: EICU collaborative research database
MIMIC-IV: Medical information mart for intensive care IV
ZPPH: Zhejiang Provincial People's Hospital
IRB: Institutional Review Board
ICU: Intensive care unit
SCr: Serum creatinine
ESKD: End-stage kidney disease
KRT: Kidney replacement therapy
RRT: Renal replacement therapy
LCTM: Latent class trajectory modeling
AIC: Akaike information criterion
BIC: Bayesian information criterion
AKI: Acute kidney injury
KDIGO: Kidney disease: improving global outcomes
RR: Risk ratio
CI: Confidence interval
BMI: Body mass index
CCI: Charlson comorbidity index
LASSO: Least absolute shrinkage and selection operator
ST: Stable
AS: Ascending
DS: Descending
HR: Hazard ratio
BUN: Blood urea nitrogen
SAE: Sepsis-associated encephalopathy
OR: Odds ratio
