# Supplementary material for: Association between serum osmolality trajectories and mortality in sepsis patients: a retrospective multi-cohort study
Source: Eur J Med Res. 2025 Dec 25;31:151. doi: 10.1186/s40001-025-03699-6 (PMC12849464; doi:10.1186/s40001-025-03699-6)

**Additional file 5. Fig. 1** Proportion of missing data for candidate variables in the MIMIC-IV, eICU-CRD, and ZPPH databases

**
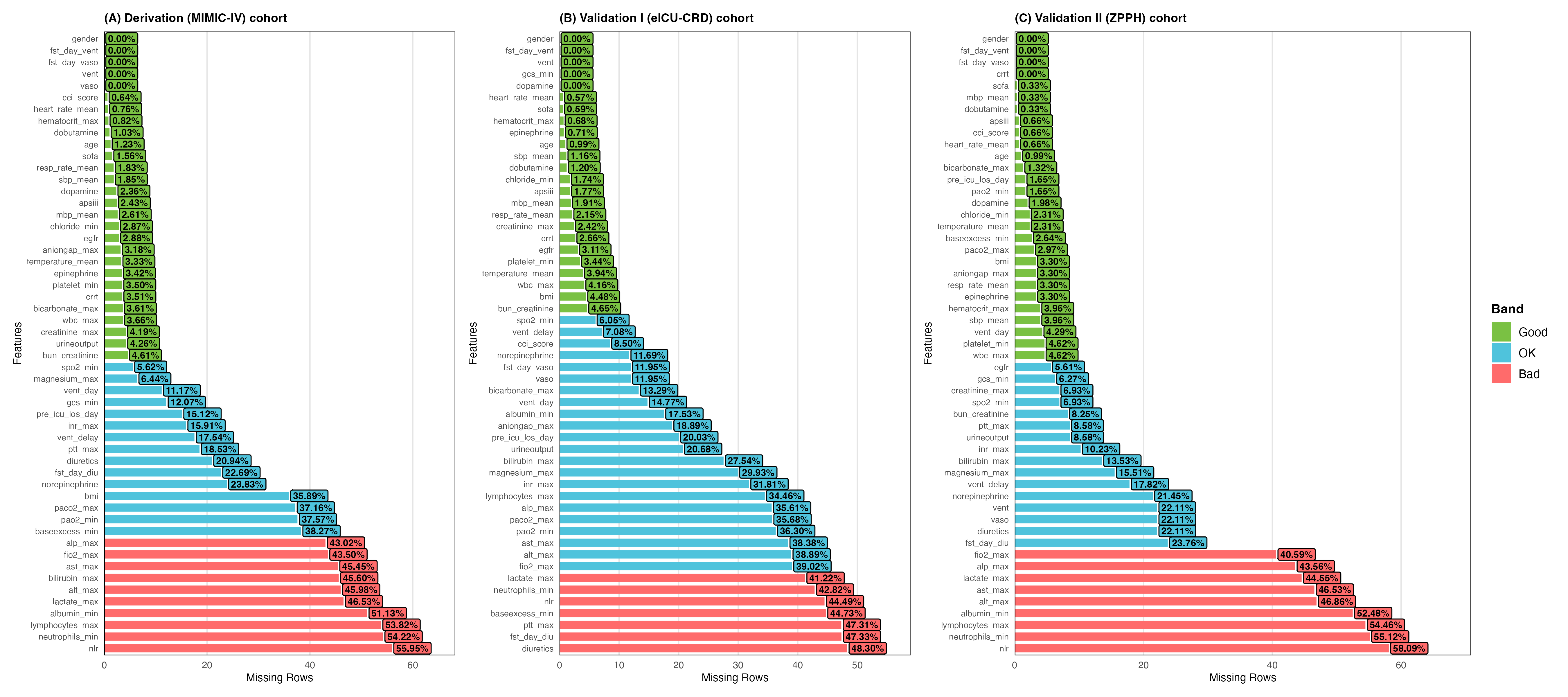
**


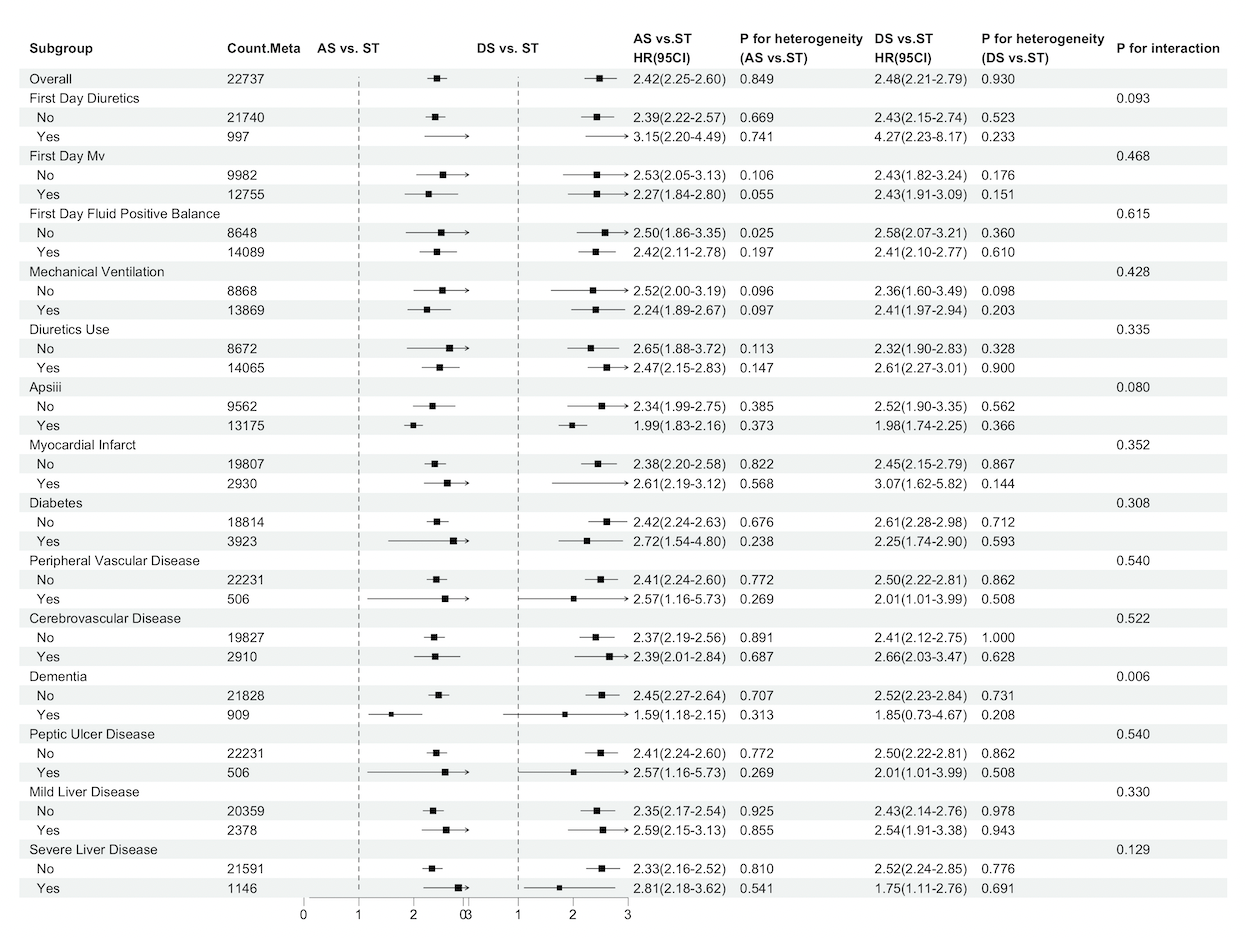
**Additional file 5. Fig. 2** Subgroup analysis of osmolality trajectory patterns and 28-Day survival: meta-analysis across MIMIC-IV and eICU-CRD databases.

**Additional file 5. Fig. 3** Correlation between calculated and biochemical serum osmolality and osmolality gap during the first 4 days from MIMIC-IV database.


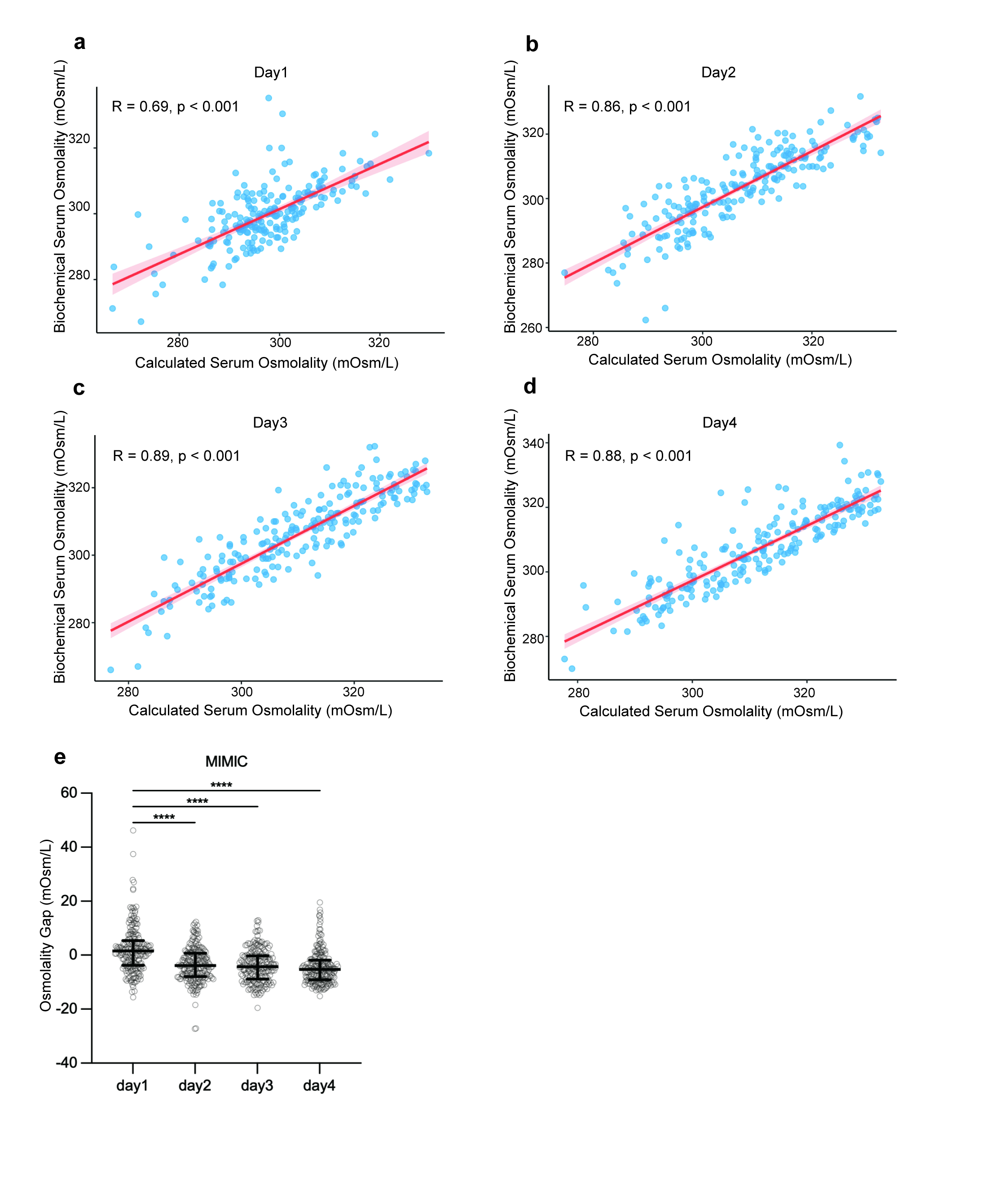


**Additional file 5. Fig. 4** Correlation between calculated and biochemical serum osmolality and osmolality gap during the first 4 days from eICU-CRD database.


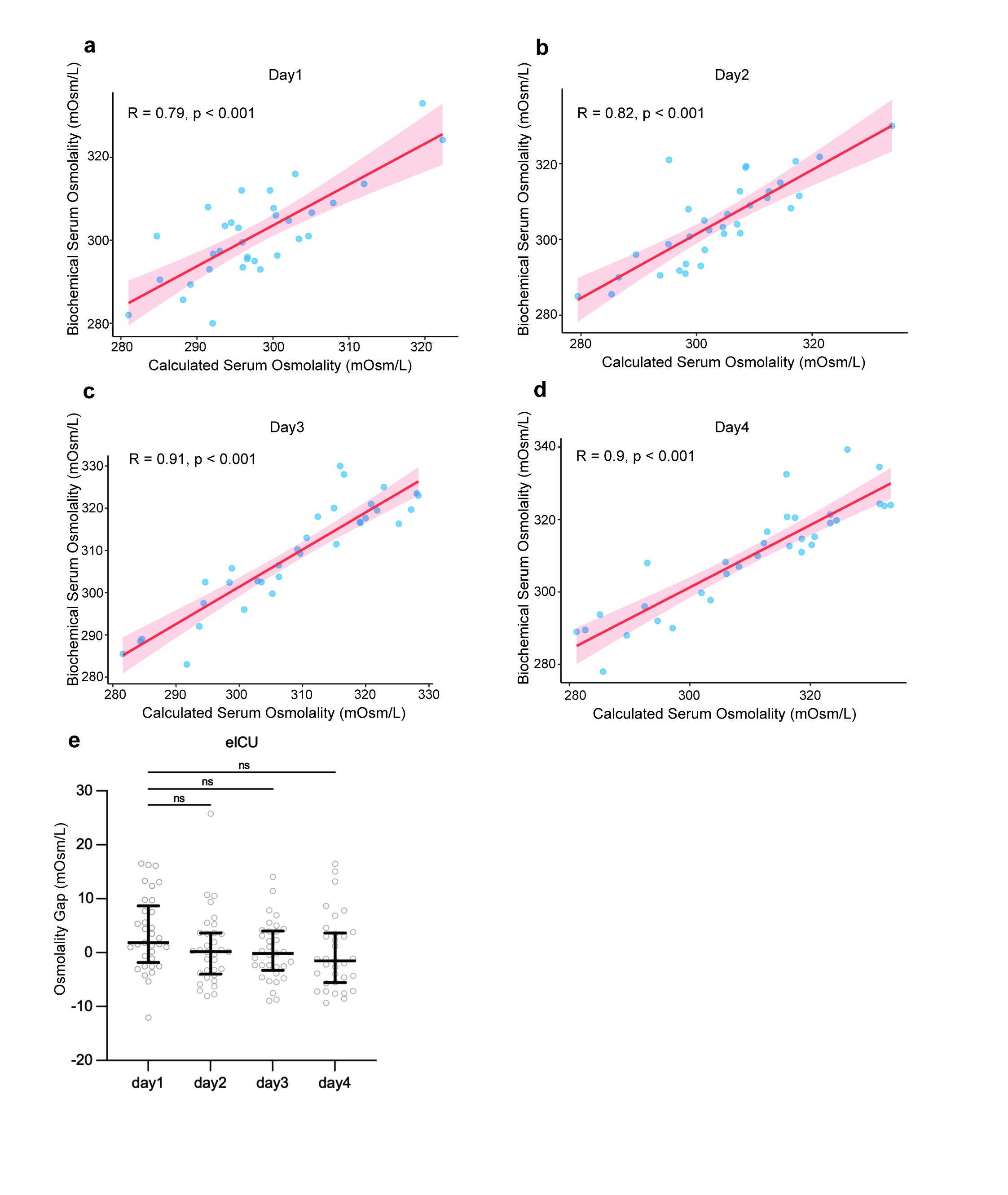


**Additional file 5. Fig. 5** Correlation between calculated and biochemical serum osmolality and osmolality gap during the first 4 days from ZPPH cohort.


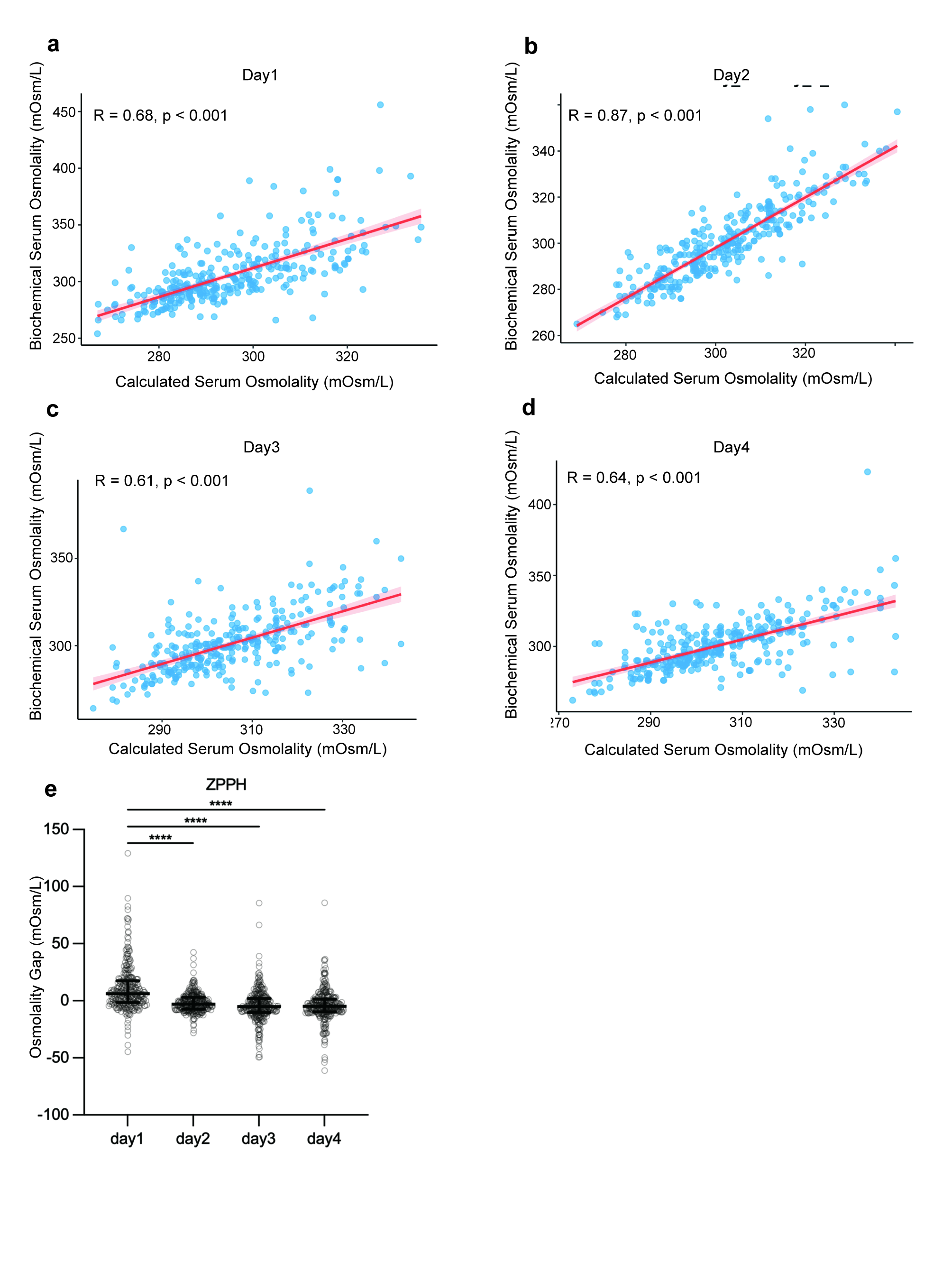

Supplement: Supplementary file 5 — Supplementary material 5. [file 40001_2025_3699_MOESM5_ESM.docx]
